# Spatial rehabilitation using virtual auditory space training paradigm in individuals with sensorineural hearing impairment

**DOI:** 10.3389/fnins.2022.1080398

**Published:** 2023-01-17

**Authors:** Kavassery Venkateswaran Nisha, Ajith Kumar Uppunda, Rakesh Trinesh Kumar

**Affiliations:** Department of Audiology, All India Institute of Speech and Hearing (AIISH), Mysore, India

**Keywords:** virtual auditory space training, localization, virtual acoustics, binaural cue processing, interaural threshold differences, perceptual ratings, spatial hearing, spatial processing

## Abstract

**Purpose:**

The present study aimed to quantify the effects of spatial training using virtual sources on a battery of spatial acuity measures in listeners with sensorineural hearing impairment (SNHI).

**Methods:**

An intervention-based time-series comparison design involving 82 participants divided into three groups was adopted. Group I (*n* = 27, SNHI-spatially trained) and group II (*n* = 25, SNHI-untrained) consisted of SNHI listeners, while group III (*n* = 30) had listeners with normal hearing (NH). The study was conducted in three phases. In the pre-training phase, all the participants underwent a comprehensive assessment of their spatial processing abilities using a battery of tests including spatial acuity in free-field and closed-field scenarios, tests for binaural processing abilities (interaural time threshold [ITD] and level difference threshold [ILD]), and subjective ratings. While spatial acuity in the free field was assessed using a loudspeaker-based localization test, the closed-field source identification test was performed using virtual stimuli delivered through headphones. The ITD and ILD thresholds were obtained using a MATLAB psychoacoustic toolbox, while the participant ratings on the spatial subsection of speech, spatial, and qualities questionnaire in Kannada were used for the subjective ratings. Group I listeners underwent virtual auditory spatial training (VAST), following pre-evaluation assessments. All tests were re-administered on the group I listeners halfway through training (mid-training evaluation phase) and after training completion (post-training evaluation phase), whereas group II underwent these tests without any training at the same time intervals.

**Results and discussion:**

Statistical analysis showed the main effect of groups in all tests at the pre-training evaluation phase, with *post hoc* comparisons that revealed group equivalency in spatial performance of both SNHI groups (groups I and II). The effect of VAST in group I was evident on all the tests, with the localization test showing the highest predictive power for capturing VAST-related changes on Fischer discriminant analysis (FDA). In contrast, group II demonstrated no changes in spatial acuity across timelines of measurements. FDA revealed increased errors in the categorization of NH as SNHI-trained at post-training evaluation compared to pre-training evaluation, as the spatial performance of the latter improved with VAST in the post-training phase.

**Conclusion:**

The study demonstrated positive outcomes of spatial training using VAST in listeners with SNHI. The utility of this training program can be extended to other clinical population with spatial auditory processing deficits such as auditory neuropathy spectrum disorder, cochlear implants, central auditory processing disorders etc.

## Introduction

Deficits in spatial hearing secondary to hearing loss have a direct bearing on day-to-day communication in listening environments (Abel et al., 2000), such as listening in noise (Kidd et al., 2005) and reverberation (Takahashi, 2009). The impact of hearing loss in listeners with sensorineural hearing impairment (SNHI) on their ability to use auditory spatial cues is readily observable on most psychoacoustical measures. Investigations on binaural processing reported poorer values in time [interaural time difference (ITD)] (Hawkins and Wightman, 1980; Kinkel et al., 1991), intensity (interaural level difference [ILD]) (Kinkel et al., 1991; Gabriel et al., 1992; Spencer et al., 2016), phase (interaural phase difference [IPD]) (Lacher-Fougère and Demany, 2005; Neher et al., 2011), and interaural cross-correlation (Gabriel et al., 1992; Spencer et al., 2016) in listeners with SNHI compared to their normal-hearing counterparts. In natural environments, changes in ITDs are usually accompanied by corresponding changes in IPDs and ILDs; however, precise control of the acoustic parameters in laboratory conditions powers the investigators to manipulate either of the cues alone or in combination, thus aiding in understanding the role of each cue (ITD, ILD, and IPD) in spatial processing.

The spatial deficits seen in SNHI listeners on the psychoacoustical measures affect their spatial performance in free-field (Best et al., 2010; van den Bogaert et al., 2011; Kuk et al., 2013; Brimijoin and Akeroyd, 2016) and closed-field scenarios (Chung et al., 2008; van Esch et al., 2013; Brimijoin and Akeroyd, 2014; Brungart et al., 2017). Apart from showing deficits in psychoacoustical measures, SNHI listeners also experience perceptual difficulties in everyday listening (Noble and Gatehouse, 2006). Spatial perception is paramount for comfortable listening in daily environments (Risoud et al., 2018), and deficits in spatial hearing places SNHI listeners at a disadvantage on a variety of tasks, including spatial navigation, speech understanding, communication in adverse listening environments (Best et al., 2010), and lowered self-confidence in their social interactions. Undoubtedly, the poor ability to localize sound accurately is a common source of frustration for SNHI listeners (Subramaniam et al., 2005). In addition, the increased spatial disability in SNHI listeners was associated with other avoidance behaviors such as the desire to escape from situations in which sounds were confusing and caused nervousness (Noble et al., 1995).

Akeroyd and Whitmer (2016) reviewed 12 studies on the localization of real sources in different quadrants (right, left, front, and back) of the acoustic field to calculate root mean square (RMS) localization error. They reported that localization/RMS error was 5° higher in SNHI listeners compared to normal hearing (NH) listeners in right-left hemifields. When considering directional acuity in the front–back dimension, mean front–back confusion rates for NH listeners were found to range from 0.1 to 5%, while those with SNHI ranged between 10 and 26% (Best et al., 2010; van den Bogaert et al., 2011). Studies exploring spatial acuity in SNHI listeners in closed-field environments using virtual sources report high front-back confusion in both SNHI and NH listeners (relative to confusions in free field), with the former exhibiting greater errors than the latter (Chung et al., 2008; van Esch et al., 2013; Brimijoin and Akeroyd, 2014; Brungart et al., 2017). Furthermore, on subjective ratings, listeners with SNHI are known to experience more serious localization difficulties that increase with the degree of HI (Noble et al., 1997; Glyde et al., 2013). Deficits in a number of peripheral and central processes including reduced audibility (Brimijoin and Akeroyd, 2016), impaired frequency selectivity (Strelcyk and Dau, 2009), poor temporal resolution, altered filtered shapes (Baker and Rosen, 2002; Bernstein and Oxenham, 2006), and increased spectral and temporal masking (Le Goff et al., 2013) can be conceived as factors for impaired spatial processing in SNHI listeners.

Although literature highlights the impact of compromised spatial acuity in human communication in listeners with SNHI, remediation programs aimed at resolving spatial deficits are surprisingly few. Some of the notable strides in enhancing spatial acuity have used hearing aids (HAs) with novel algorithms for auditory spatial coding (Drennan et al., 2005), gain settings (Keidser et al., 2011), bilateral synchronization (Johnson et al., 2017), and listening configurations (van den Bogaert et al., 2006; Neher et al., 2009). Although theoretically enhancing temporal, spectral, and intensity cues aiding directional perception should be possible using novel spatial processing algorithms in HAs, studies involving the localization ability of HA users have shown poor-to-mixed results (refer to reviews—Denk et al., 2019; Zheng et al., 2022). Reports suggest slight improvements in localization ability after a period of acclimatization in SNHI listeners (Noble and Byrne, 1990; Drennan et al., 2005) however, contrary evidence on decreased localization performance after HAs usage is also available abundantly (Byrne and Noble, 1998; van den Bogaert et al., 2011; Akeroyd, 2014). A number of factors such as the design of HA, degree of loss, cognition, age of the participant, testing condition (aided or unaided, noise or quiet, open or closed earmolds), and test material (speech, noises with various bandwidths, center frequencies, spectral slopes, and real-world sounds such as telephone ring) could have influenced these results. Akeroyd and Whitmer (2016) reviewed 36 studies that compared directional acuity of unaided and bilaterally aided hearing-impaired (mostly mild to severe sloping and older hearing-impaired adults) listeners and reported only a slight 1° difference between the aided and unaided scores (the RMS error: RMS values were 12° for unaided listening and 13° for aided listening). Although within-subject variability was seen between aided and unaided conditions with the differences reaching statistical significance in a few reports (Keidser et al., 2009; van den Bogaert et al., 2011), contrary evidence is also available (Drennan et al., 2005; Best et al., 2010; Brungart et al., 2014). Only four of 36 reviewed studies (11.11%) showed a benefit of aiding of at least 1°, whereas more than 20 studies (55.55%) showed a deficit of aiding of at least 1° and nine (=25%) reports showed a deficit of 3° or more. Taken together with the multiplicity of differences between studies (HA styles, test materials, and conditions) in Akeroyd and Whitmer's (2016) review, the results can be treated to be more or less representative of the effect of aiding on spatial perception. The use of HAs (as seen in studies earlier) can negatively affect spatial perception as they reduce the HRTF cues and also distort ITD and ILD cues.

Alternatively, minimal improvements in spatial performance are reported when spatial training programs use loudspeakers in free field (Tyler et al., 2010; Kuk et al., 2014) or interaural difference training (Wright and Fitzgerald, 2001; Rowan and Lutman, 2006; Spierer et al., 2007; Zhang and Wright, 2007; Ortiz and Wright, 2009) under closed field (headphones), although these improvements were not clinically or statistically significant. The clinical utility of training-related remedial programs in auditory spatial perception is limited by a number of factors, such as those related to study design (small sample sizes, heterogeneous outcome measures, inconsistent use of control groups, and limits of generalization) as well as those related to technical aspects such as length of the training programs and the cost–benefit ratio.

All the research efforts in documenting the effects of spatial deficits can be productive only if promising intervention strategies are devised. In addition to the minimal spatial acuity improvements reported in SNHI listeners consequent to training, a lot of issues related to study design and technical aspects question their utility in day-to-day practice. It is unknown whether everyday localization accuracy can be facilitated by training and whether improvements identified in a laboratory setting can be sustained and generalized.

The present study is intervention-based research that investigated the effect of virtual auditory space training (VAST) (Nisha and Kumar, 2017, 2018) on spatial acuity in listeners with SNHI. VAST is a novel paradigm that relies on auralization techniques to synthesize spatial percepts called virtual acoustic stimuli, which cause an illusionary effect of natural sound-field localization within the head (King et al., 2001). The virtual stimuli are constructed by superposing the target stimuli with the non-individualized HRTFs (refer to methods for stimulus generation), that enriches the stimuli with important spatial cues such as the ITDs, ILDs, spectral, and HRTFs. Thus, generated virtual stimuli are played back within the head using headphones in a systematically graded order of spatial difficulty (refer to methods for the hierarchy of stimulus presentation) and the listener is trained to achieve mastery in each level under self-supervision (refer to methods for detailed training procedure). The use of the VAST paradigm is proven to be as effective as free-field spatial training using loudspeakers in fine-tuning spatial skills of NH listeners (Nisha and Kumar, 2022) and has promising implications on cortical re-organization (Nisha and Kumar, 2019a). In addition, the use of virtual stimuli identical to those used in the VAST paradigm has been effective in documenting the effects of SNHI on cortical processing (Nisha and Kumar, 2019a,b), the effects of maturational and aging-related changes across life-span (Nisha et al., 2023), and the musical training effects (Nisha et al., 2022). The present study aimed to validate the efficacy of the VAST paradigm in resolving spatial perception deficits in SNHI listeners. Specifically, the objectives of the study were to document the spatial processing abilities in SNHI and compare the same with NH listeners using spatial acuity measures in the free field and closed field, binaural cue processing, and subjective ratings. The study additionally aimed to compare the pre-, mid-, and post-training performance of SNHI listeners on the above psychoacoustic spatial measures at different timelines as a function of VAST.

## Methods

### Participants

A total of 82 participants in the age range of 35–55 years were recruited for the present study, and they were divided into three groups. Groups I and II consisted of listeners with mild to moderate (Katz, 2015) flat SNHI (Pittman and Stelmachowicz, 2003). While the group I (*n* = 27, 17 males, 10 females, M_age_ = 43.8 ± 10.44 years SD) consisted of individuals with SNHI who underwent spatial training (VAST), group II consisted of individuals with SNHI who did not receive spatial training (*n* = 25, 12 males, 13 females, M_age_ = 43.7 ± 5.92 years SD). In addition, group III (*n* = 30, 17 males, 13 females, M_age_ = 38.55 ± 3.25 years SD) had participants with normal peripheral hearing sensitivity. The sample size considered in the study was calculated using G^*^Power version 3.1.9.4 (Faul et al., 2007) based on the effect size reported by Kuk et al. (2014) on spatial training in SNHI listeners. Although the sample size calculated was only 8 participants in each group for an effect size of 1.39, a higher sample size of at least 25 was considered for each group in the present study. In contrast to Kuk et al.'s (2014) study, where the SNHI participants who underwent spatial training also used HAs, the current study was performed on SNHI listeners who did not use HAs. The induction of SNHI listeners who had no previous exposure of HAs helped us to avoid potential limitations of HAs on auditory spatial processing (refer to the “Introduction” Section). The sample size used by us (group I = 27, group II = 25, and group III = 30) was, therefore, deemed appropriate for measuring changes due to VAST.

**Figure 1:**
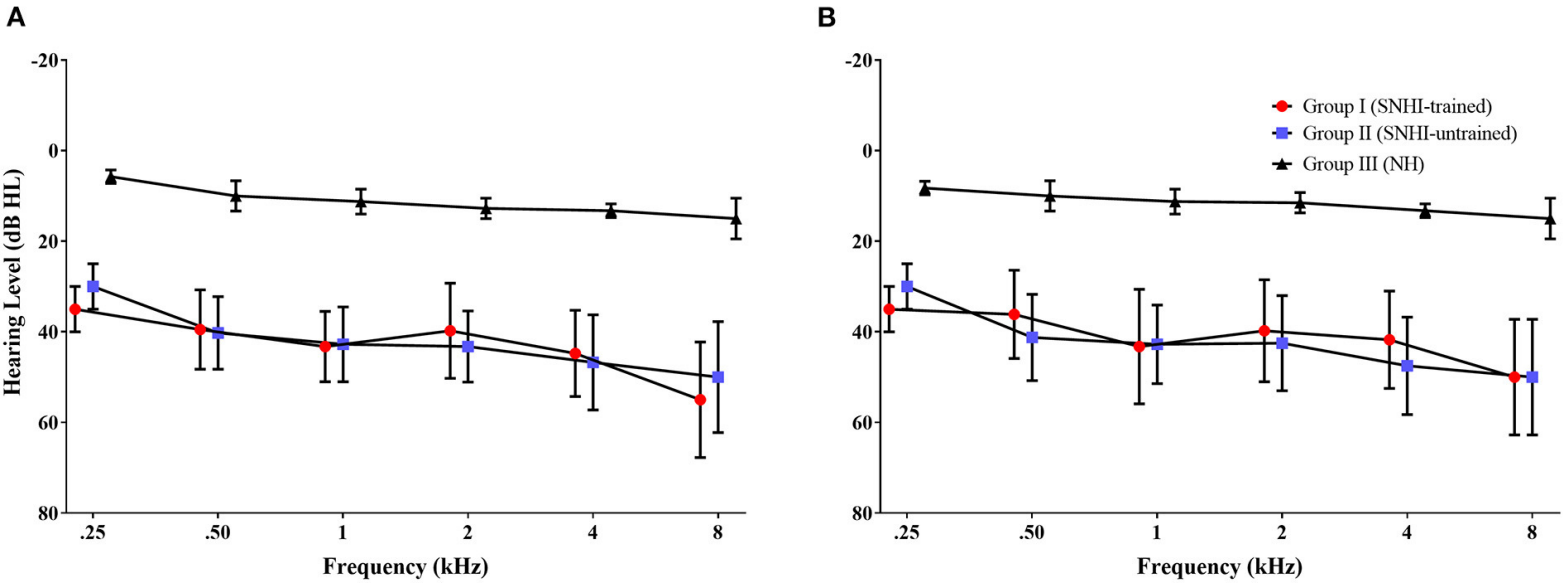
The mean hearing thresholds of participants in each group across audiometric frequency range for **(A)** right ear and **(B)** left ear. The error bar represents ± 1 SD.

**Figure 2:**
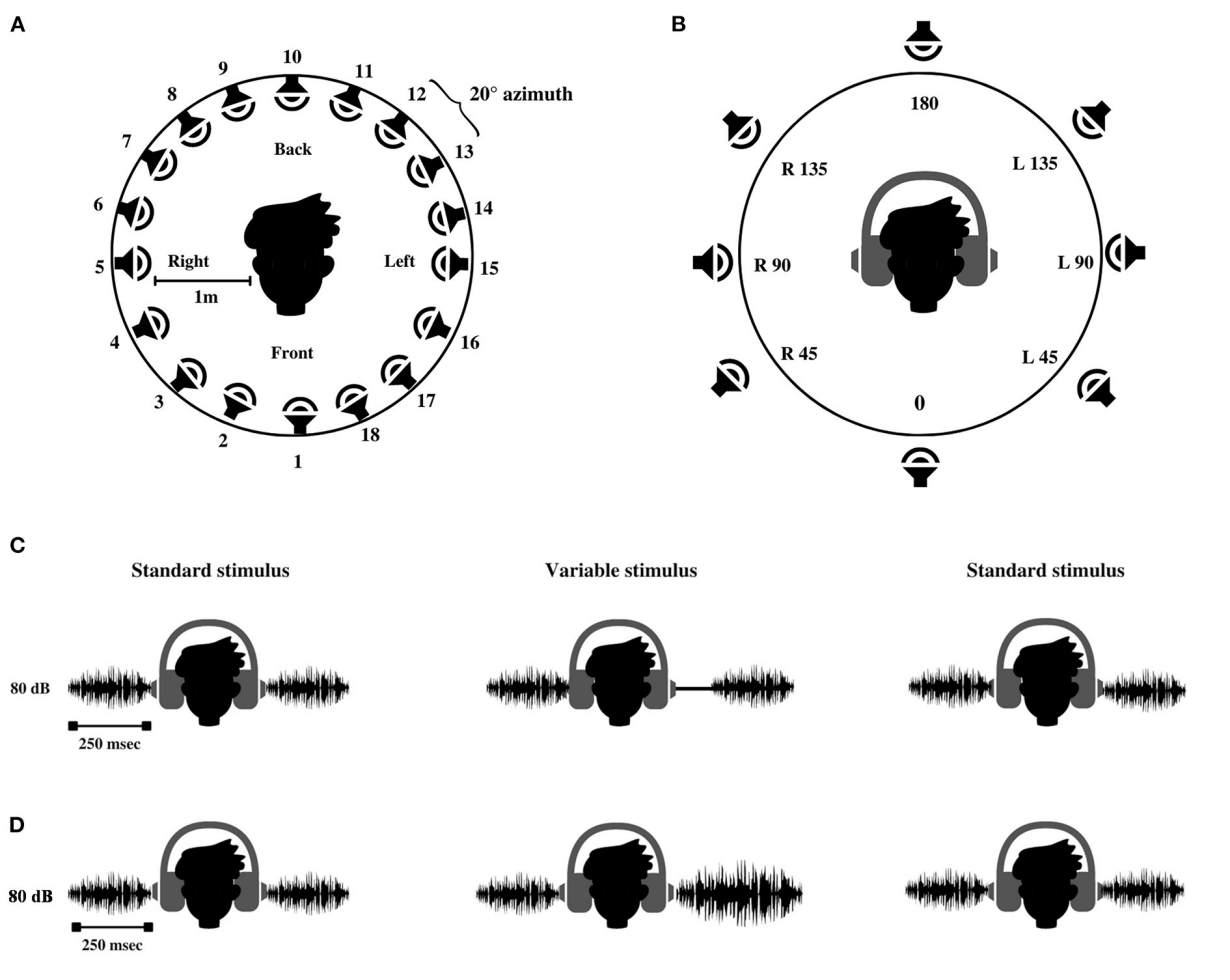
Schematic representation of **(A)** loudspeakers setup used for testing spatial acuity in free field. **(B)** Interface used for stimulus and response acquisition in virtual auditory space identification (VASI) test. The alphanumerical code represents the location of spatial percept created within the head. 0°-At the midline front, 180°-At the midline back, R45–45° azimuth toward the right ear; R90–90° azimuth toward the right ear, R135–135° azimuth toward the right ear; L45–45° azimuth toward the left ear, L90–90° azimuth toward the left ear, and L135–135° azimuth toward the left ear. **(C)** Interaural time difference test and **(D)** interaural level difference test.

All the participants were subjected to a detailed case history to rule out any external and middle ear pathology. Pure tone audiometry was conducted on all participants where both the air conduction (250–8,000 Hz) and bone conduction thresholds (250–4,000 Hz) were obtained at the octave frequencies using the modified Hughson and Westlake procedure given by Carhart and Jerger (1959). The air conduction and bone conduction thresholds of these participants were tested using a Piano inventis audiometer (Inventis, Padova, Italy) by routing stimulus through Telephonics TDH 39 earphones (Telephonics, Farmingdale, NY, USA) and B71 bone vibrator (RadioEar, Kimmetrics, Smithsburg, MD, USA). Based on four frequency pure tone AC thresholds (pure tone average (PTA) obtained as an average of thresholds at 500, 1,000, 2,000, and 4,000 Hz), participants with mild to moderate SNHI formed groups I and II. The SNHI was further confirmed by the absence of otoacoustic emissions and acoustic reflexes (both ipsilateral and contralateral reflex) recorded using standard recording protocol in Otodynamics ILO V6 DP Echoport (Otodynamics Ltd., Hatfield, Herts, UK) and Inventis Clarinet (Inventis Inc., Padova, Italy) instruments, respectively. Among these two groups, the former received formal spatial training using VAST and the latter served as the control group that did not receive any spatial training. Participants with NH sensitivity, i.e., PTA <25 dB HL were selected for group III. Figure 1 shows the thresholds for (Figure 1A) right and (Figure 2B) left ears across the groups. The group differences in the thresholds were verified using one-way ANOVA [right ear: *F*_(2,79)_ = 171.31, *p* < 0.001; left ear: *F*_(2,79)_ = 121.82, *p* < 0.001], followed by Bonferroni comparisons which showed that the thresholds of the two SNHI groups were similar (right ear: *p* = 0.75; left ear: *p* = 0.35) and significantly higher (right and left ears: *p* < 0.001) than the NH group.

Participants with any neurological and cognitive deficits were excluded from the study. Mini-Mental State Examination (MMSE) (Folstein et al., 1983) translated into Indian English (Milman et al., 2018) was administered to rule out any pathological cognitive decline (all participants scored more than 24 points). Group equivalency in cognition across groups was also cross-checked statistically from the results of one-way ANOVA, which showed no main effect of the group [*F*_(2,79)_ = 1.09, *p* = 0.34] on MMSE scores. In addition, the influence of musical training and aptitude in participants of the study was ruled out based on informal interviews (for any formal history of musical training) and cutoff scores ( ≤ 18) on mini-Profile of Music Perception Skills (mini-PROMS, Zentner and Strauss, 2017). The results of one-way ANOVA for mini-PROM verified that there were no differences in musical aptitude across the three groups [*F*_(2,79)_ = 1.19, *p* = 0.31].

Written consent was obtained from all the participants, which confirmed their willingness to participate in the research study. The study was approved by the institutional core committee on research and adhered to the institutional ethical guidelines of bio-behavioral research involving human subjects (Venkatesan, 2009) under the reference number SH/CDN/ARF-AUD-4/2018-19.

### Research design

A mixed design (which includes both between-subjects design and within-subjects design) (Schiavetti and Metz, 2006) based on the intervention-control model was used. Standard group comparison (SNHI vs. NH) was adopted to study the spatial processing differences between the groups (between-subjects design), while a time-series (pre-, mid-, and post-training) design was used to evaluate the effect of VAST on the auditory spatial performance of SNHI listeners (within the subject design).

### Procedure

The study was conducted in three phases, i.e., pre-training, training, and post-training phase.

#### Phase I: Pre-training evaluation phase

In the pre-training phase, the spatial acuity of all listeners was assessed using a test battery comprising three psychoacoustical measures and one subjective measure as discussed in the following sections. The stimulus presentation level was maintained constant at 80 dB SPL for all the psychoacoustical tests employed in this study.

##### Test of spatial acuity in free field (localization test)

White noise bursts of 250 ms (inclusive of 5 ms rise and fall time) were generated using the AUX viewer software (mono, 32 bits, 44,100 sampling rate), calibrated to the output level of 80 dB SPL (using sound level meter; Bruel and Kjaer 2270, Naerem, Denmark), and used as stimuli for the test of spatial acuity in free field (localization test). The stimuli were loaded on a personal computer and were assigned to 18 audio tracks using the Cubase software (Steinberg Media Technologies GmbH, Hamburg). One of the 18 tracks delivered the stimuli to the corresponding loudspeakers *via* Aurora mixer (Lynx Studio Technology Inc., California, USA). The 18 loudspeakers (Genelec 8020B BI-amplified monitoring system, Finland) were placed in a concentric circle with a spacing of 20° from each other covering a complete spatial field spanning 360° azimuth, as shown in Figure 2A. The 18 loudspeaker array with 20° separation used in the current study is in accordance with the localization setups used for spatial studies on hearing-impaired listeners (Lorenzi et al., 1999; Drennan et al., 2005; Keidser et al., 2009).

The participant was asked to sit comfortably on a chair placed at a distance of 1 m in a center of the loudspeaker array in the localization chamber (semi-anechoic). After the delivery of the stimulus, the participant was asked to judge the location of the loudspeaker that emitted the sound and respond by writing the number corresponding to the loudspeaker. The test started with a pilot trial where 10 random presentations of the stimuli were given to make the participant familiarize to the task. In the testing phase, the stimuli were presented five times to each of the 18 speakers (in random order based on the track sequence in Cubase), and the participant was asked to respond by writing the speaker's number on a response sheet. Once the response to a particular trial was completed, the participant was asked to indicate its completion with a thumbs-up sign to the experimenter. The inter-trial interval for stimulus presentation depended on the response time of the participant. The next stimulus was presented only after the participant registered his response to the previous stimuli. The test was terminated after a total of 90 presentations (18 loudspeaker locations × 5 repetitions) and was completed in ~15 min.

The responses were then entered in the user interface built for spatial error analysis in paradigm experimenter builder software (Perception Research Systems, 2007). A program written in a Python script running in the background of the interface recorded the target and the response location. The overall localization errors (RMS error) were computed in accordance with the formula given by Rakerd and Hartmann (1986). RMS error represents the root mean square of actual response deviation (in ° azimuth) from the target location. This was done for all the participants across the three groups.

##### Test of spatial acuity in the closed field (virtual auditory space identification test)

VASI comprised of presentation of acoustic stimulus under the headphone at the target azimuths, which mimicked the free-field environment presentation. Although the stimuli were presented through the circumaural headphones (Sennheiser HD 280 PRO, Wedemark, Germany), the use of appropriate equalization techniques provided good azimuth replication that was comparable with the spatial hearing performance of individuals with NH in free-field environments (Pralong and Carlile, 1996; Zhong and Xie, 2013). Virtual percepts in the VASI test were created by convolving 250 ms white noise bursts with a non-individualized HRTF obtained from the sound lab (Slab3d) database. The sound lab (Slab 3D) version 6.7.3 (Spatial Auditory Displays lab, 2012) was used to control the generation of the virtual percepts in eight target locations: Midline front: 0° azimuth, midline back: 180° azimuth, and 45°, 90°, and 135° azimuth to the right and left. All the stimuli had constant elevation (0° azimuth) and distance (1 m). The virtual stimuli were synthesized to be identical to the free-field stimuli in terms of overall level (80 dB SPL) and duration (250 ms). The generated stimuli were calibrated to a level of 80 dB SPL using the sound level meter (Bruel and Kjaer 2270, Naerem, Denmark). The synthesized stimuli were loaded into the paradigm software, which controlled stimulus delivery and response acquisition using a graphical user interface, as shown in Figure 2B.

To allow for familiarization to VASI stimuli, the participants were encouraged to use practice runs. A dummy head (Figure 2B) was displayed on the monitor screen during these runs. The participant was instructed to use the mouse to click on each virtual location (no more than five trials per stimulus), and the corresponding virtual sound was emitted. In the testing phase, the eight stimuli were presented ten times at each location in random order. The order of presentation is randomized using a designated function in the stimulus characteristics window of the paradigm software. The participant was asked to attend to the virtual stimuli and click the mouse pointer on the position of the dummy head (Figure 2B), corresponding to the perceived location in the head. No feedback was given during the familiarization and testing phase. The test involved a presentation of a total of 80 virtual stimuli (eight loudspeaker locations × ten repetitions) to all the participants and was completed in ~15 min. After the completion of the experiment, the data corresponding to the target and the response locations stored in the output (excel) file was derived. The data comprising the VASI accuracy scores for each virtual location and overall VASI score (aggregate score of all eight virtual locations) were computed using a confusion matrix script (Gnanateja, 2014) in MATLAB version 2021b (The MathWorks Inc., Natick, MA, USA).

##### Binaural processing (ITD and ILD thresholds)

The binaural processing abilities of the participants in the study were assessed using ITD and ILD thresholds. The test of ITD and ILD involved the presentation of two identical signals to both ears, with one ear receiving the signal slightly earlier or at a higher intensity relative to the other. The lowest intensity level at which a person reports the difference in intensity between the two ears is considered the ILD threshold. In the ITD test, the smallest time delay that a person can identify is considered a threshold for ILD. The difference in the time of arrival or the intensity between the two ears created lateralization of the stimuli toward one ear, which is to be detected by the participant.

The binaural abilities in the current study were assessed using a psychoacoustic toolbox (Soranzo and Grassi, 2014) implemented in MATLAB version 2021b (The MathWorks Inc., Natick, MA, USA). The ITD and ILD thresholds were measured in the three-interval forced-choice method. A two-down one-up staircase procedure was followed, which converged at 70.7% psychometric function (Levitt, 1971). Among the three stimuli in one trial, two were standard stimuli, and one was the variable stimulus. White noise bursts (250 ms, stereo, 16 bit, 44,100 sampling frequency, 80 dB SPL) similar in terms of binaural intensity and time of arrival, producing a midline sensation, were designated as standard stimuli. The variable stimuli were similar to the standard stimuli, except that it produced lateralization to the right ear due to inherent delay (introduced in the left ear) and increased intensity in one channel (increased intensity in one ear in the ILD test). The participants were instructed to compare the intensity of the signal between the two ears and report the interval in which the sound was lateralized to the right ear. The variable stimuli either led (ITD) or were heard louder (ILD) in the right ear, as shown in Figures 2C, D.

The time delay and intensity given in variable intervals are changed adaptively based on the response of the participant. The starting level of the delay for variable stimulus in the ITD test was 30 ms, which decreased by half when the participant recorded correct judgments in two successive trials (or) doubled when the participant made an incorrect judgment. For the test of ILD, starting level of variable stimuli was 20 dB higher in the right ear. The level of signal changed successively by a step size of 2 dB as the test progressed. The level was reduced by 2 dB when the participant recorded two successive correct responses and increased by 2 dB on registering an incorrect response. The test was terminated after 10 reversals, and the last four reversals were averaged to calculate the ITD and ILD thresholds. The ITD and ILD thresholds were tabulated and subjected to statistical analyses.

#### Subjective ratings (Spatial sub-section of Spatial, Qualities, and Hearing Questionnaire in Kannada)

Participants rated the perceptual difficulties in spatial orientation using the Spatial sub-section of SSQ (Gatehouse and Noble, 2004), translated to Kannada (SSQ-K) (Shetty et al., 2019). This list contained 17 items that are administered on an 11-point rating scale, where 0 represents the minimal ability and 10 represents the complete ability to locate the sound source accurately.

#### Phase II: Spatial training and mid-training evaluation phase

Participants in group I (SNHI-trained) underwent virtual acoustic space (VAST) using a hierarchy of graded VAS stimuli. The spatial training was performed using VAS stimuli as it facilitated definite simulation of spatial (azimuth) location within the head (Wenzel et al., 1993; Hartmann and Wittenberg, 1996). The use of VAS stimuli for spatial training allowed us to have systematic control on varying the levels of spatial perception difficulty, which were introduced using important source lateralization cues, namely, the length/duration of the signal and the number of locations. In addition, VAST enhanced the practicality of the spatial training paradigm as its implementation required only minimal equipment, which was easily portable, and the participants could undergo spatial training at home as well.

**Figure 3:**
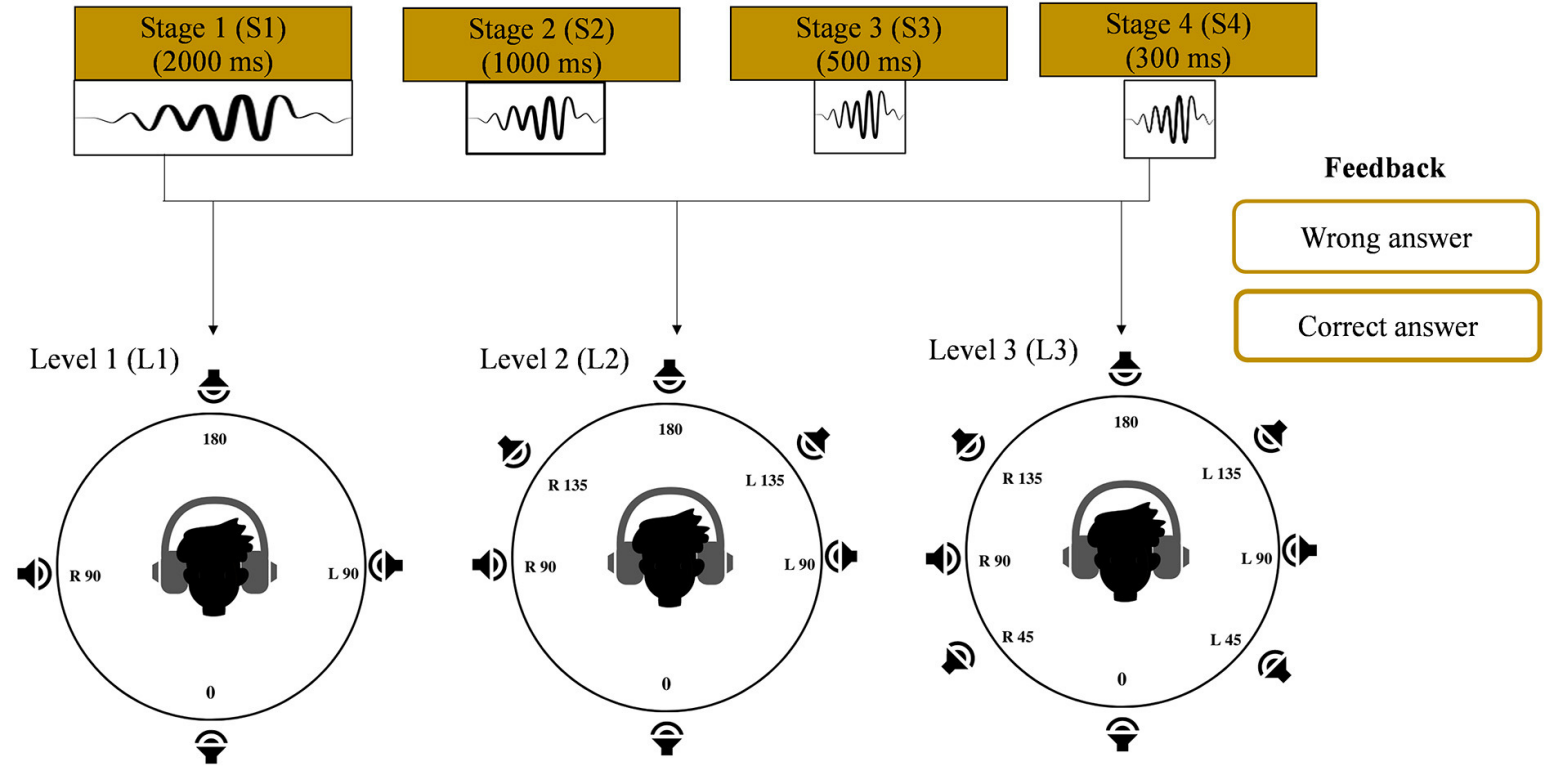
Hierarchy of stimulus (duration and number of virtual locations parameters) presentation in the training phase of the study. Progression from left to right represents easy to difficult conditions. Feedback given during the training is also shown.

The VAST paradigm used in the current study was adapted from Kuk et al. (2014). The VAST paradigm was proven to be effective in resolving front-back confusion in NH listeners (Nisha and Kumar, 2017, 2022), and thus its application in SNHI listeners was conceptualized in the present study. The complexity of VAS stimuli varied adaptively in terms of their durations (stages: 2,000, 1,000, 500, and 300 ms) and the number of locations (levels 4, 6, and 8), as shown in Figure 3. Irving and Moore (2011) showed that a longer stimulus is easier to localize and easier to train than a shorter stimulus. It is also easier to judge the spatial locations of virtual sources that are distant apart than those that are closely spaced (Carlile et al., 2016). Training commenced from the easiest level (S_1_L_1_: 2,000 ms, four locations) and accurate judgments were counted. The VASI accuracy at each level was calculated, based on which they progressed to the next difficult level based on 70% VASI accuracy criteria. The most challenging level (S_4_L_3_) was stimuli in stage 4 with a duration of 300 ms and eight locations.

##### VAST paradigm

The user interface for VAST was built using the Paradigm experimental builder software. Two interfaces were separately designed for familiarization and training modules. In each of these interfaces, a display of a dummy head with four, six, and eight locations corresponding to levels 1, 2, and 3, respectively, is configured, as shown in Figure 3. The training was carried out in two modules. Module I was a familiarization task, while module II was a training task. In the familiarization module, the participants were encouraged to get familiar with the VAS stimuli (not more than 10 trials per stimulus) using a practice run. In this run, a graphical interface consisting of a dummy head with varying VAS locations (4 in level 1, 6 in level 2, and 8 in level 3) was displayed on the monitor. The participants were asked to click the mouse pointer on the position of the dummy head, and the corresponding VAS sound file was played. In the training module, the stimuli were played randomly and the participants were instructed to cautiously attend to the stimuli and click the mouse pointer on the position of the dummy head (Figure 3) corresponding to the perceived location in the head. Each VAS stimulus was randomly presented 7 times, thus making a total of 28 (4 locations × 7 repetitions) presentations for level 1, 42 (6 locations × 7 repetitions) presentations for level 2, and 56 (eight locations × seven repetitions) presentations for level 3 in each stage (stage 1: 2,000 ms; stage 2: 1,000 ms; stage 3: 500 ms; and stage 4: 300 ms). Once the participant registered his response through the mouse click, corrective feedback on the response was given. The correct responses were acknowledged, while the incorrect responses were compared with the correct location. 70% criterion was set up to progress from each level. The total VAST paradigm was completed in 5–8 sessions (30 min each), depending on the rate of learning and mastery obtained by the participant.

Halfway through training, the spatial skills of group I participants were re-assessed using all the tests conducted in the pre-training phase. This assessment provided an opportunity to understand the time course/pattern of spatial learning in group I participants who underwent VAST. All the tests were also conducted in group II participants (SNHI-untrained), which constituted the second evaluation (at a similar timeline as the post-training evaluation in group I) in them. Following this evaluation, the participants in group I completed the remaining stages of training.

#### Phase III: Post-training evaluation phase

Immediately after the completion of training (0–5 days post-training), the spatial test battery was re-administered to the group I participants. The spatial tests were also administered on group II participants (SNHI-untrained), which constituted the third evaluation (at a similar timeline as the post-training evaluation in group I) in them.

### Statistical analysis

The data obtained from all the tests were subjected to statistical analyses using the IBM Statistical Package for the Social Sciences (SPSS) version 25 software (IBM Corp., Armonk, NY, USA). Shapiro–Wilk test of normality was employed to check if the data follow the normal or non-normal distribution. For the data that followed a normal distribution, multivariate analysis of variance (MANOVA) and follow-up pairwise comparisons using independent *t*-tests (with Bonferroni's correction) were carried out for each measure of spatial acuity. These tests were conducted to compare the performance of SNHI (groups I and II) and NH (group III) participants. However, the Mann–Whitney *U*-test was performed for data that did not adhere to normal distribution. For analyzing the effect of training across evaluation phases in group I and II participants, within-subject tests of repeated measure ANOVA, followed by Bonferroni's test or Friedman test and then by Dunn–Bonferroni's test were carried out for normal and non-normal data distributions, respectively.

In addition, to explore the impact of VAST on various behavioral measures of spatial acuity in group I (SNHI-trained) and to compare the same with the spatial performance in group II (SNHI-untrained) and NH participants (group III), Fischer's discriminant analysis (FDA) was done. A default mathematical operation (Di = a + b1 x 1 + b2 x 2 +…+ bnxn; Di = predicted discriminant score; a = a constant, x = predictor; and b = discriminant coefficient) run in SPSS version 25 for group categorization was employed in the study. The main purpose of discriminant function (DF) analysis in this study was for group segregation and identification of the optimal spatial measure (RMS error, VASI scores, ITD and ILD thresholds, and spatial subsection of SSQ-K scores) that best predicts VAST-related changes. The FDA was performed for each measurement phase separately (pre-, mid-, and post-evaluation), while the error in classification at each phase is also reported.

## Results

### Comparison of the spatial performance of listeners with sensorineural hearing impairment and normal hearing sensitivity

**Figure 4:**
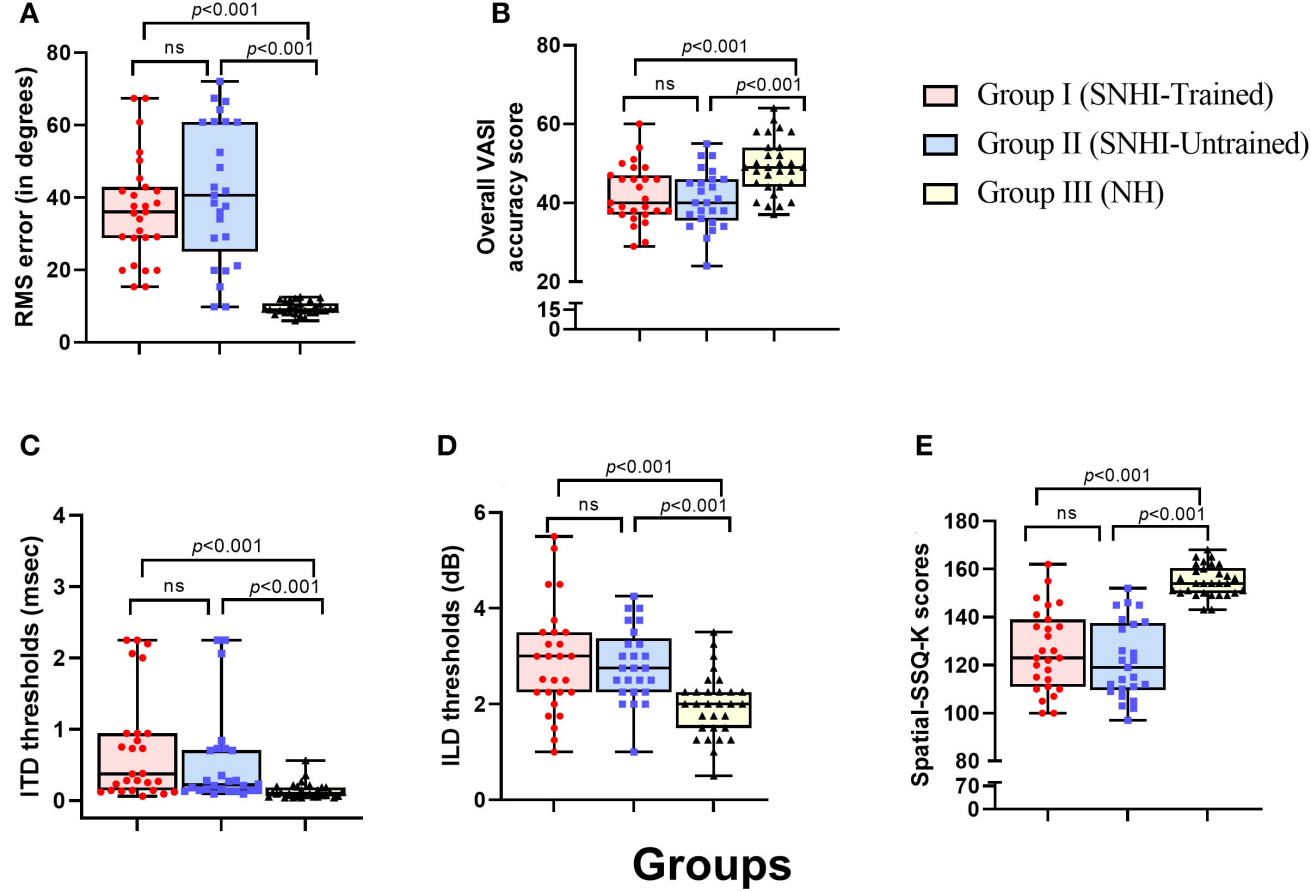
Comparison of spatial acuity across groups in **(A)** test of spatial acuity in free field, **(B)** test of spatial acuity in closed-field, **(C)** interaural time difference thresholds, **(D)** interaural level difference thresholds, and **(E)** subjective ratings on spatial sub-section of SSQ-K. The horizontal line in each box plot at center represents the median, while the “+” sign indicates the mean for each test. The box area corresponds to interquartile range, while the error bar indicates interquartile deviation. The results of Bonferonni comparisons are also indicated.

The descriptive statistics showing the median and mean along with the interquartile range for all the measures of spatial acuity measures used in the study; localization (RMS) error scores, overall VASI scores, ITD and ILD thresholds, and perceptual SSQ ratings across the three groups (group I: SNHI-trained; group II: SNHI-untrained; and group III: NH listeners) obtained at pre-training evaluation revealed that SNHI participants (groups I and II) demonstrated spatial acuity deficits compared to NH, as reflected in Figure 4. Results of the MANOVA test showed the main effect of the group for the localization test [*F*_(2,79)_ = 46.78, *p* < 0.001, $\eta_{\text{p}}^{2}$ = 0.54], VASI test [*F*_(2,79)_ = 10.51, *p* < 0.001, $\eta_{\text{p}}^{2}$ = 0.21], ILD [*F*_(2,79)_ = 9.93, *p* < 0.001, $\eta_{\text{p}}^{2}$ = 0.20], and spatial sub-section of SSQ-K [*F*_(2,79)_ = 49.11, *p* < 0.001, $\eta_{\text{p}}^{2}$ = 0.55]. The *post hoc* comparisons using the Bonferroni test indicated that RMS errors in the free-field localization test of group I (36.63°± 14.37 SD) were similar (*p* > 0.05) to group II (40.96°± 17.25 SD), although both groups I and II registered significantly higher (*p* < 0.001) RMS errors than NH listeners (9.41°± 1.73). A similar trend was also observed in VASI, ILD, and spatial subsection of SSQ-K tests with group equivalency of both SNHI groups (groups I and II), who demonstrated significantly poorer (*p* < 0.001) spatial acuity scores (i.e., groups I and II had lower VASI and SSQ scores and higher ILD thresholds) compared to the NH group. The non-parametric Kruskal–Wallis test also revealed a significant main effect of the group for ITD [*H* (2) = 27.25, *p* ≤ 0.001, η^2^(H) = 0.27]. Upon *post hoc* analyses using Dunn–Bonferroni pairwise comparisons, participants with SNHI (groups I and II) were shown to have significantly higher (*p* < 0.001) ITD thresholds compared to individuals with NH sensitivity indicative of binaural temporal cue processing deficits in them. Although SNHI listeners registered poorer spatial acuity than NH listeners on all spatial acuity tests, the performance between the former two groups was similar, indicating their group equivalency.

### Effect of VAST on spatial acuity measures

**Figure 5:**
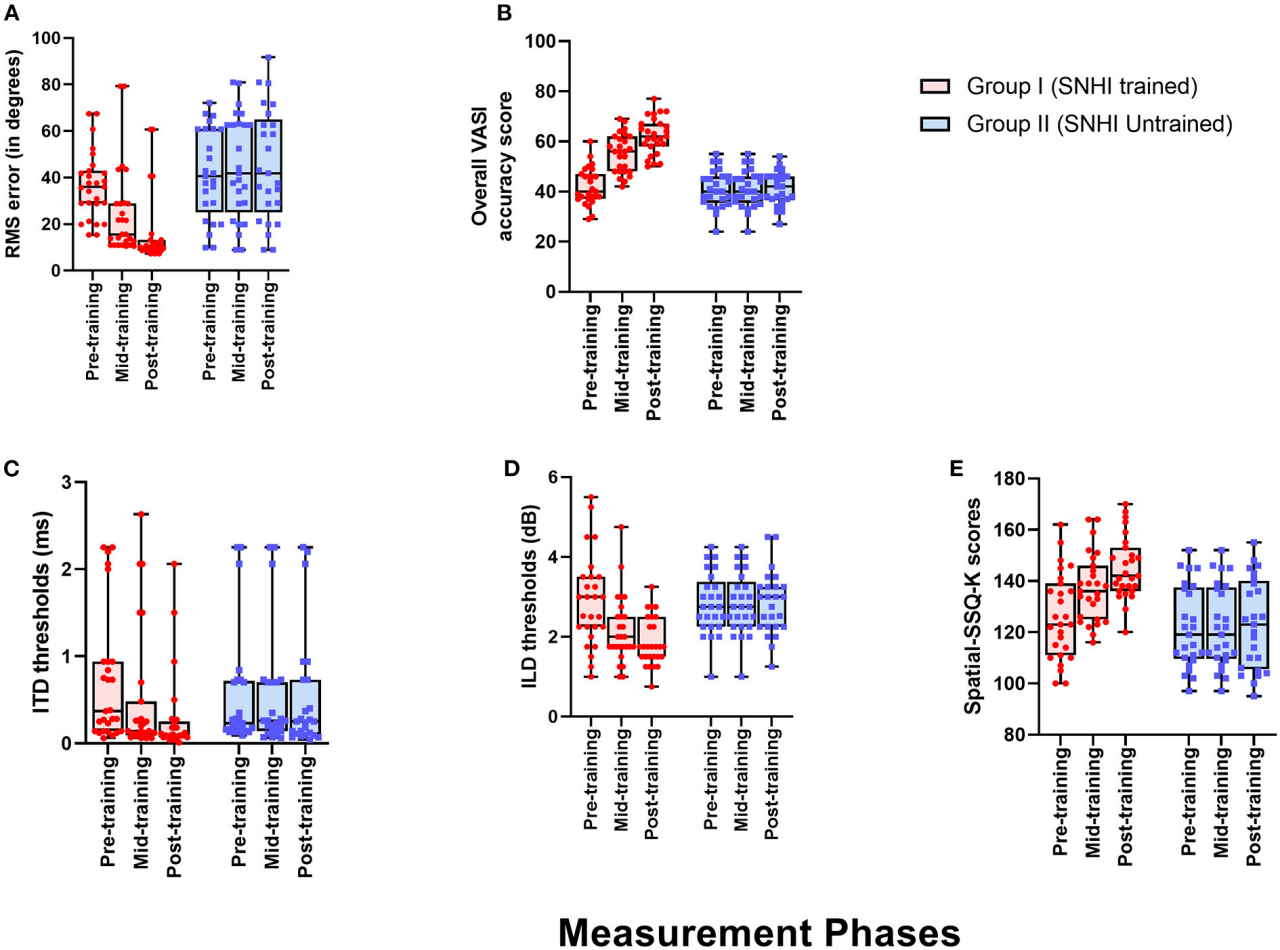
Comparison of spatial acuity of group I (SNHI-trained) and group II (SNHI-untrained) participants as a function of VAST in **(A)** test of spatial acuity in free field, **(B)** test of spatial acuity in closed-field, **(C)** interaural time difference thresholds, **(D)** interaural level difference thresholds, and **(E)** subjective ratings. The horizontal line in each box plot at center represents the median, while the “+” sign indicates the mean for each test. The box area corresponds to interquartile range, while the error bar indicates interquartile deviation. The Bonferroni comparisons between pre-, mid-, and post-evaluations were given for only group I participants, as similar comparisons for group II yielded no difference between evaluations.

To evaluate the effect of VAST on the spatial acuity of SNHI listeners, the pre-, mid-, and post-training scores of group I along with two evaluations (at time-intervals equivalent of pre- and post-training evaluations) of group II were compared, with the median and mean along with the interquartile range is reflected in Figures 5A–E.

The spatial performance of group I (SNHI-trained) participants who underwent VAST improved as a function of training in all the measures, as reflected in the improvement of median/mean scores and reduction in variability with progression in spatial training, as shown in Figure 5. No noticeable change was seen in both median/mean in the group II (SNHI-untrained) participants, who did not undergo any formal training. The statistical significance of such differences explored using repeated measure ANOVA (3 evaluation phases) or Friedman test for each measure separately, along with their corresponding effect sizes in groups I and II, is shown in Table 1. The significant main effect of the evaluation phase was observed for all the spatial acuity measures in group I (SNHI-trained), while no main effect of the evaluation phase (*p* > 0.05) was seen for SNHI-untrained (Table 1), who showed no observable changes in their spatial acuity across evaluations.

**Table 1:** Results of Friedman test for main effect of evaluation phase and follow-up adjusted Bonferroni's pairwise comparisons for SNHI-trained and SNHI-untrained groups in each test of spatial processing.

	**Group I (SNHI trained)**				**Group II (SNHI untrained)**
**Tests**	**Main effect of evaluation phase**	**Bonferroni comparisons**			**Main effect of evaluation phase**
		**Pre-training**	**Mid-training**	**Post-training**	
RMS Error	$\chi_{\left(2\right)}^{2}$ = 48.67, *p* < 0.001, Kendall's W = 0.90	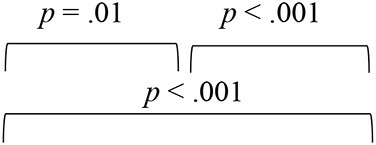			*F*_(2,48)_ = 1.90, *p* = 0.31
VASI	*F*_(2,52)_ = 105.02, *p* < 0.001, $\eta_{\text{p}}^{2}$ = 0.80	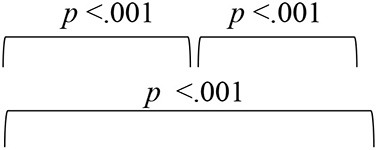			*F*_(2,48)_ = 0.03, *p* = 0.97
ITD	$\chi_{\left(2\right)}^{2}$ = 38.34, *p* < 0.001, Kendall's W = 0.70	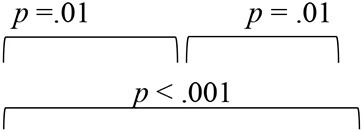			$\chi_{\left(2\right)}^{2}$ = 3.58, *p* = 0.17
ILD	$\chi_{\left(2\right)}^{2}$ = 38.00, *p* < 0.001, Kendall's W = 0.70	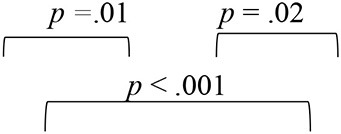			*F*_(2,48)_ = 0.01, *p* = 0.99
SSQ	*F*_(2,52)_ = 89.31, *p* < 0.001, $\eta_{\text{p}}^{2}$ = 0.78	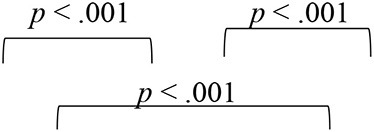			*F*_(2,48)_ = 0.61, *p* = 0.55

**Figure 6:**
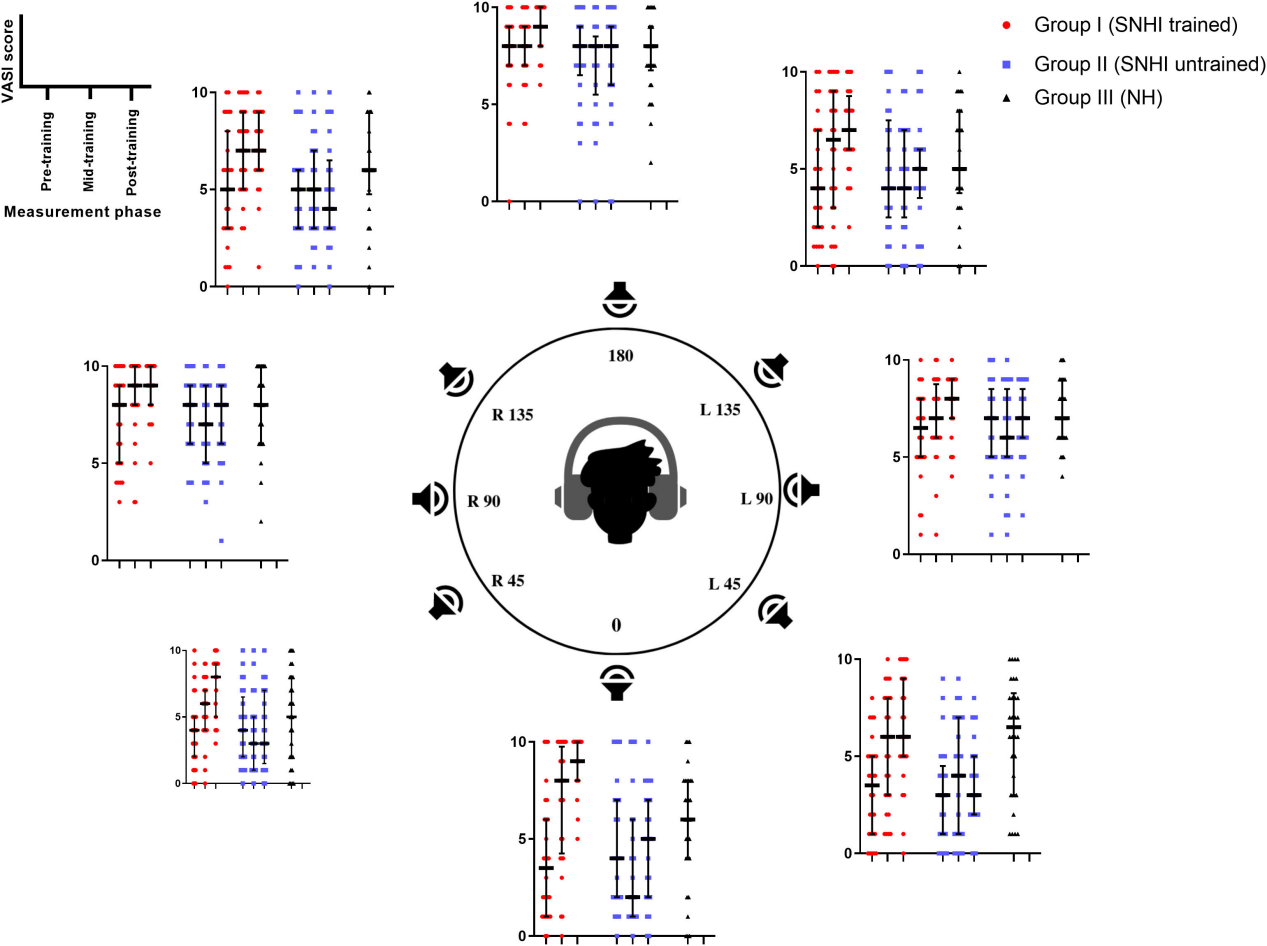
Comparison of VASI scores of group I (SNHI-trained) and group II (SNHI-untrained) participants as a function of VAST across in virtual location. The inner dummy head panel represents eight VAS locations used in the study, while the outer panels denote VASI scores of each participant corresponding to VAS location mentioned in the inner panel at three evaluation phases (pre-, mid-, and post-training). The horizontal line in each box plot at center represents the median, while the “+” sign indicates the mean for each test. The box area corresponds to interquartile range, while the error bar indicates interquartile deviation. The VASI of group III (NH listeners) is given only for comparison purpose. Maximum score per location is 10.

The group I participants who underwent VAST showed improved spatial acuity not only on the overall VASI, but the spatial training benefits were also evident on the location-wise scores, as shown in Figure 6. The SNHI-trained group demonstrated improved VASI scores at the mid- and post-training evaluations, relative to the pre-training evaluation phase, while no changes in virtual location perception were seen in the SNHI-untrained group. Scores obtained by NH listeners are also depicted for comparison purposes. The improvement of virtual location identification seen in the SNHI-trained group was statistically significant on the Friedman test (along with Dunn–Bonferroni *post hoc*), while no main effect of the evaluation phase was seen in the SNHI-untrained group, as shown in Table 2. On closer visual inspection of Figure 6, VASI scores at each location in the SNHI-trained group not only improved across evaluation phases but also outperformed NH, in the post-training phase at all virtual locations.

**Table 2:** Results of Friedman test for main effect of evaluation phase and follow-up adjusted Bonferroni's pairwise comparisons for SNHI-trained and SNHI-untrained groups at each virtual location in VASI test.

	**Group I (SNHI trained)**				**Group II (SNHI untrained)**
**Virtual location**	**Main effect of evaluation phase**	**Bonferroni comparisons**			**Main effect of evaluation phase**
		**Pre-training**	**Mid-training**	**Post-training**	
R45	$\chi_{\left(2\right)}^{2}$ = 24.74, *p* < 0.001, Kendall's W = 0.46	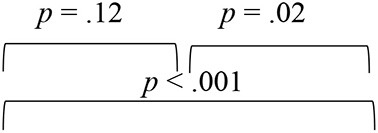			$\chi_{\left(2\right)}^{2}$ =0.27, *p* = 0.87
R90	$\chi_{\left(2\right)}^{2}$ = 6.71, *p* = 0.04, Kendall's W = 0.12	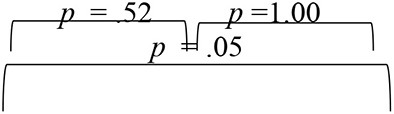			$\chi_{\left(2\right)}^{2}$ = 1.16, *p* = 0.45
R135	$\chi_{\left(2\right)}^{2}$ = 6.15, *p* = 0.05, Kendall's W = 0.11	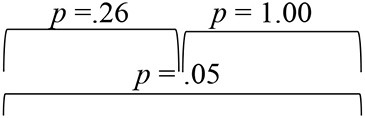			$\chi_{\left(2\right)}^{2}$ = 0.02, *p* = 0.98
180	$\chi_{\left(2\right)}^{2}$ = 9.78, *p* = 0.01, Kendall's W = 0.18	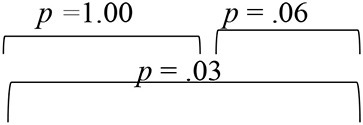			$\chi_{\left(2\right)}^{2}$ = 0.53, *p* = 0.77
L135	$\chi_{\left(2\right)}^{2}$ = 17.43, *p* < 0.001, Kendall's W = 0.32	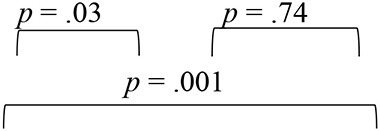			$\chi_{\left(2\right)}^{2}$ = 0.51, *p* = 0.77
L90	$\chi_{\left(2\right)}^{2}$ = 6.81, *p* = 0.04, Kendall's W = 0.31	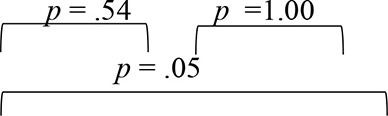			$\chi_{\left(2\right)}^{2}$ = 4.08, *p* = 0.13
L45	$\chi_{\left(2\right)}^{2}$ = 9.78, *p* = 0.01, Kendall's W = 0.18	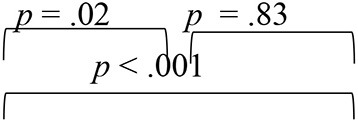			$\chi_{\left(2\right)}^{2}$ = 2.02, *p* = 0.36
0	$\chi_{\left(2\right)}^{2}$ = 30.02, *p* < 0.001, Kendall's W = 0.18	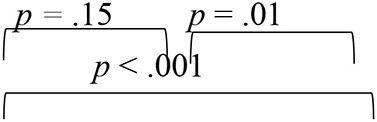			$\chi_{\left(2\right)}^{2}$ = 0.53, *p* = 0.77

The italic text correspond to significance value ‘p' or test statistic, as used conventionally in reporting.

### Identifying the optimal test for measuring VAST-related changes in spatial processing in SNHI

The discriminant functional analysis generated two DFs that effectively categorized VAST-related changes in spatial acuity, combinedly for all the tests considered in the study. While DF_1_ was statistically the most robust function (*p* < 0.001) for the group segregation based on spatial processing abilities, DF2 was not significant. The extent of variability explained by DF_1_ and DF_2_ across measurement phases is shown in Table 3. The variability explained by DF_2_ was relatively less, ranging between 1.50 (pre-training) and 29.5 (post-training).

**Table 3:** Eigen values, Wilk's lambda (λ), and percentage of variance for the standardized discriminant functions (DF_1_ and DF_2_) in pre-, mid-, and post-training evaluation phases.

**Evaluation phase**	**Discriminant function**	**Eigen value**	**% of variance**	**Wilk's lambda (λ)**	**Chi-square test**
Pre-training	1	2.45	98.50	0.28	$\chi_{\left(10\right)}^{2}$ *=* 98.36, *p* < 0.001
	2	0.04	1.50	0.96	$\chi_{\left(4\right)}^{2}$ = *2.91, p* = 0.57
Mid-training	1	2.12	79.00	0.21	$\chi_{\left(10\right)}^{2}$ *=* 121.87, *p* < 0.001
	2	0.56	21.00	0.64	$\chi_{\left(4\right)}^{2}$ *= 34.32, p* = 0.06
Post-training	1	2.37	70.50	0.15	$\chi_{\left(10\right)}^{2}$ *=* 146.67, *p* < 0.001
	2	0.99	29.50	0.50	$\chi_{\left(4\right)}^{2}$ *= 53.10, p* = 0.14

The italic text correspond to significance value ‘p' or test statistic, as used conventionally in reporting.

Table 4 shows the relative contribution (weights) of each test in group membership (SNHI-trained, SNHI-untrained, and NH) on DF_1_ across the three evaluation phases (pre-, mid-, and post-training). The coefficient with large absolute values corresponds to RMS error (free-field localization test measure for spatial acuity) for all the evaluation phases, indicative of the higher predictive power of this metric for group categorization.

**Table 4:** Contribution (weights) of auditory spatial measures for group membership on discriminant function 1 (DF_1_).

**Predictor variable/tests**	**Pre-training**	**Mid-training**	**Post-training**
RMS error (free-field test)	0.66	0.56	0.54
Overall VASI scores	−0.12	−0.06	−0.43
ITD thresholds	0.11	0.05	0.07
ILD thresholds	0.19	0.36	0.27
Spatial-subsection of SSQ-K scores	−0.57	−0.67	−0.39

Based on the weights (Table 4), the canonical DF_1_ obtained in the study for each evaluation phase is summarized below:

Pre-training DF_1_: (0.66 × RMS error) + (0.11 × ITD thresholds) + (0.19 × ILD thresholds)–(0.12 × overall VASI)–(0.57 × SSQ-K).

Mid-training DF_1_: (0.56 × RMS error) + (0.05 × ITD thresholds) + (0.36 × ILD thresholds)–(0.06 × overall VASI)–(0.67 × SSQ-K).

Post-training DF_1_: (0.54 × RMS error) + (0.07 × ITD thresholds) + (0.27 × ILD thresholds)–(0.43 × overall VASI)–(0.39 × SSQ-K).

**Figure 7:**
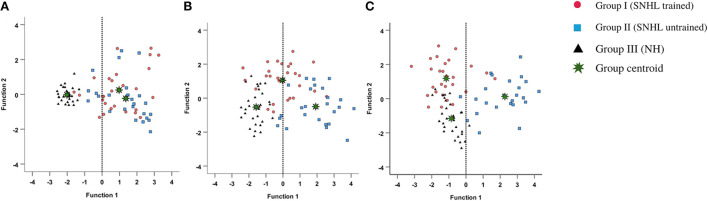
Grouping participants based on canonical discriminant scores derived for behavioral measures of spatial acuity at **(A)** pre-, **(B)** mid-, and **(C)** post-training evaluation phases. The red circles, blue squares, and black triangles correspond to discriminant scores of group I (SNHI-trained), group II (SNHI-untrained), and group III (NH) participants, respectively. The categorization of the groups is evident on the significant function, i.e., DF1. Function 2 is merely used for plotting the abscissa values, and group classification is not elaborately discussed as this function was not significant.

The analyses of the DF1 function across evaluation phases (as summarized by weightages in equations earlier and Table 4) identified RMS error as the most sensitive metric that can capture spatial perception benefits derived through VAST in SNHI listeners. The combined group plot obtained using the results of FDA was plotted using DF_1_ on abscissa and DF_2_ on the ordinate, and a cluster of classification values of spatial performance across tests for different groups is shown in Figure 7. The cluster of classification values for all the groups was calculated by multiplying the standardized canonical DF coefficient by the test results of each individual on the five associated spatial measures and summing these products. Thus, calculated mean and individual scores for each group (group centroids) on the two DFs are shown in Figure 7. On visual inspection of the combined group plot, it can be seen that DF1 helped in the effective categorization of the differences in auditory spatial performance between the groups at all three phases of evaluation.

The combined group (Figure 7) plot also depicted the emergence of two distinct clusters of spatial performance on DF_1_. In the pre-training evaluation phase (Figure 7A), while the symbols corresponding to SNHI groups (SNHI-trained: red circles; SNHI-untrained: blue squares) were concentrated on the right side of the DF_1_, the symbols circles (black triangles) denoting the NH group emerged as a distinct cluster on the left side of the function. The marked disparity seen in the distribution of the two clusters (two SNHI groups relative to the NH group) at the pre-training phase (Figure 7A) became less apparent at the mid-training phase (Figure 7B), wherein a gradual shift in spatial acuity performance of SNHI-trained (red circles) due to VAST is materialized (seen as a shift in predicted scores toward NH). At the mid-evaluation phase (Figure 7B), the distribution of clusters involving red circles (SNHI-trained) moved slightly toward the right (relative to the concentration of red symbols in the pre-training condition) on the DF_1_. This cluster movement toward the NH group (black triangles) indicated the initial realization of benefits derived from VAST on behavioral spatial acuity measures. It is also important to note that the SNHI-untrained group did not show any visible movement in the mid-training phase, as opposed to the SNHI-trained indicative of no spatial performance changes in them. The separation of the clusters (SNHI-trained and NH) became nearly extinct separation on DF_1_ at the post-training phase (Figure 7C). At the post-training evaluation phase (Figure 7C), the cluster distribution of SNHI-trained (red circles) advanced further to the right of DF_1_, causing the superimposition of red and black symbols. This camouflaging of the cluster distributions on the DF_1_ in the post-training evaluation phase signals the materialization of positive outcomes of VAST in group I (SNHI-trained) participants.

Furthermore, the errors in the prediction of group membership based on classification results of DF analysis also revealed that differentiating the groups based on DF_1_ caused no confusion in classifying NH into the same group (predicted membership of NH was similar to original NH) at pre-training condition, as shown in Table 5. This delineation was not readily apparent between the SNHI-trained and SNHI-untrained groups, with a consistent overlap occurring between them. Only 55.66% of SNHI-trained and 60.00% of SNHI-untrained were correctly classified, accounting for the error of 44.34 and 40%, respectively. This finding showed that at pre-training conditions, the spatial acuity of the trained and untrained groups was similar, making the group membership prediction difficult for the algorithm. At the mid-training phase, the categorization error of the SNHI-trained decreased to 29.63%, while that of SNHI-untrained dropped to 20.0%, indicating the change in auditory spatial performance of the SNHI-trained group consequent to the VAST paradigm, which in turn successfully segregated the spatial performance of the former group from the latter. Complementary to the same, the accuracy of group prediction also increased to 70.37% and 80.0% for the SNHI-trained and SNHI-untrained groups, respectively. This trend in decreased group classification errors and increased prediction accuracy was persistent in the post-training phase as well, with chances of error in grouping reduced to 22.20 and 16% for SNHI-trained and SNHI-untrained groups. However, the errors of classifying SNHI-trained as NH increased in the post-training evaluation, with 6 NH being wrongly classified as SNHI-trained (as opposed to only 2 NH misclassified as SNHI-trained in mid-evaluation and none in pre-training), indicative of spatial performance in few participants in the SNHI-trained group nearing spatial abilities of NH participants.

**Table 5:** Classification results for groups in pre-, mid-, and post-training evaluation phases.

**Evaluation phase**	**Groups**	**Original count**			**Percentage classified (%)**		
		**Group I (SNHI-trained)**	**Group II (SNHI-untrained)**	**Group III (NH)**	**Group I (SNHI-trained)**	**Group II (SNHI-untrained)**	**Group III (NH)**
Pre-training (73.22% of original grouped cases correctly classified)	Group I (SNHI-trained)	15	8	0	55.66	32.00	0.00
	Group II (SNHI-untrained)	10	15	0	37.00	60.00	0.00
	Group III (NH)	2	2	30	7.44	8.00	100.00
	Total	27	25	30	100.00	100.00	100.00
Mid-training (81.77% of original grouped cases correctly classified)	Group I (SNHI-trained)	19	2	2	70.37	8.00	6.67
	Group II (SNHI-untrained)	2	20	0	7.41	80.00	0.00
	Group III (NH)	6	3	28	22.22	12.00	93.33
	Total	27	25	30	100.0	100.0	100.00
Post-training (80.55% of original grouped cases correctly classified)	Group I (SNHI-trained)	21	1	6	77.78	4.0	20.00
	Group II (SNHI-untrained)	2	21	0	7.41	84.0	0.00
	Group III (NH)	4	3	24	14.81	12.0	80.00
	Total	27	25	30	100.0	100.0	100.00

## Discussion

In an intervention-based research design, the present study investigated the application of VAST in resolving auditory spatial deficits in SNHI listeners, apart from comparing the same to NH listeners. The findings of MANOVA and Kruskal–Wallis *H*-test indicated a significant main effect of group, with the consequent *post hoc* tests revealing higher spatial resolution skills in NH listeners, in terms of both precision (lower localization errors and lower ITD and ILD thresholds) and accuracy (higher VASI and spatial sub-section of SSQ-K scores) compared to both SNHI groups (SNHI-trained and SNHI-untrained). In contrast, the participants with SNHI who were either in VAST-trained or VAST-untrained groups demonstrated similarity in their spatial performance (equally poorer skills), suggestive of group equivalency prior to spatial training. The spatial acuity deficits seen in SNHI listeners (SNHI-trained and SNHI-untrained) in the present study are in consensus with literature accounts on different spatial measures such as localization (Häusler et al., 1983; van den Bogaert et al., 2006), lateralization (Kubo et al., 1998; Spierer et al., 2007), and binaural cue processing (Koehnke et al., 1995; Smith-Olinde et al., 1998; Spencer et al., 2016). Deficits in a number of peripheral processes, such as impaired frequency selectivity (Turner et al., 1999; Bernstein and Oxenham, 2006; Hopkins and Moore, 2011), temporal resolution (Arehart, 1998; Bianchi et al., 2016), and altered auditory filter shapes (Glasberg and Moore, 1986; Dubno and Dirks, 1989) in individuals with SNHI can be conceived as factors that account for these group differences.

The results of the within-subject analysis in the SNHI-trained group showed significant improvements in all spatial tests at mid-evaluation and further refinement of the same at post-training evaluation (Table 2), indicative of positive outcomes of VAST. The present study demonstrated that the effect of the VAST paradigm was not restricted to just the trained stimuli but was generalizable to other tasks such as localization of real sources, thresholds of ILD and ITD, and perceptual ratings (Figures 5A–E). While the improvements in VASI score reflect stimulus-specific learning, i.e., learning the task for which one is trained, improvements on other spatial tasks signal perceptual learning (Wright and Fitzgerald, 2001). Thus, the paradigm used in the study seems to validate the process of supervised learning in which related perceptual networks calibrate each other in a goal-directed way (Knudsen, 1984), as recorded in several reports on perceptual learning (Zahorik et al., 2006; Wright et al., 2010). The generalization effect reported in the current study is also supported by the observation of Ortiz and Wright (2009) who found that ITD/ILD training effects were generalized to temporal acuity (GAP detection) skills (not implicitly trained) apart from improving ITD/ILD thresholds. The success derived from the VAST is further strengthened by findings in group II participants, who continued to demonstrate spatial deficits at the mid- and post-training evaluation. This benefit derived from VAST can be explained by the modified auditory adaptation–feedback model of spatial processing proposed by Mendonça (2014).

Auditory adaptation–feedback model in its original form was constructed to explain auditory spatial adaptability to altered signals. Drawing parallels to the current spatial training paradigm, i.e., VAST, altered signals in the original model are equated to distorted (due to consequence of SNHI) direction-dependent inputs (binaural and spectral cues). These cues, which lack precise binaural/spectral information, are further combined in the peripheral auditory system to estimate the virtual source position. Owing to degraded inputs at the peripheral auditory system in SNHI listeners, an incorrect space percept of the virtual source is formed. This incorrect virtual auditory space percept is then followed by feedback. As approached in VAST, the feedback given was the corrective response feedback of virtual sound location. The correct responses are acknowledged, while the incorrect responses are compared and contrasted with the correct item. Tyler et al. (2010) also reported success when participants were allowed to compare loudspeaker locations. In VAST, the feedback is compared to the original virtual auditory space percept. If no differences are found (i.e., in the case of correct response), the original virtual sound percept is strengthened. If the feedback is substantially different from the percept, then a new cue combination rule (set of ITD, ILD, and spectral cues) is created. On multiple repetitions of the feedback, the new cue combination gets further strengthened, and a new spatial percept is created. A forward–backward loop between the perceptual mechanisms involved in original virtual space perception and feedback is created. We postulate that spatial learning occurs precisely from this loop. The application of feedback in perceptual learning is advocated in learning theory, which supports the idea that best learning occurs when listeners are motivated (Amitay et al., 2010). When the listeners sense that the task is impossible to succeed, or when they feel the limited challenge in the task, their motivation for learning diminishes. However, changing the stimulus difficulty (length and number of virtual locations) adaptively in VAST ensured that the motivation level of listeners during training was maintained high.

The benefits derived from the spatial learning loop can be influenced by other non-acoustic perceptual factors (Andéol et al., 2015), including its ability to maintain the motivation level of the listener (Amitay et al., 2010). In accordance with the same, the VAST paradigm started with an easier stimulus (longer duration), and difficulty was adaptively adjusted based on the listeners' performance level. Furthermore, as mentioned earlier, visual and auditory feedback was provided during the training (listen and compare). This was done to encourage self-correction and help participants know which directions required more attention. This feedback along with a structured hierarchy of stimulus presentation forms the core strength of VAST and serves as a primary basis for the realization of benefits derived from it.

The spatial abilities of listeners with SNHI who underwent VAST (group I) were also compared to NH listeners (group III) to measure the extent of spatial learning in them using FDA. Discriminant analyses showed that the RMS error measure of the free-field localization test had the highest predictive power for group categorization in all the evaluation phases (Table 4), suggestive of its robust sensitivity to VAST-related benefits. Specifically, the benefit derived due to VAST in reducing localization error in the current study (36.02° in pre-training to 10.11° in post-training evaluation, Figure 5A) is relatively higher than the improvements seen in other psychoacoustic (overall VASI, Figure 5B, and binaural processing scores, Figures 5C, D) and subjective (spatial-sub-section of SSQ, Figure 5E) measures used in the study. The improvement seen in localization abilities secondary to VAST reported in the present study is similar to the earlier reports in the literature by Tyler et al. (2010), who demonstrated that the pre-training localization score consisted of an average RMS error of 24°, while the post-training score was 17° RMS error. Further combined group plot (Figure 7) of FDA showed distinct clusters of SNHI listeners (SNHI-trained and SNHI-untrained) from NH listeners in the pre-training evaluation phase, although there was considerable overlapping of the former two groups (SNHI-trained and SNHI-untrained) with a relatively higher prediction error between these groups (Table 5). In the mid-training followed by post-training, the error in classification decreased between the SNHI groups (SNHI-trained and SNHI-untrained), showing the improved scores as a function of VAST in the trained group, making it easier for group membership prediction. Complimentary to the earlier, there was a rightward movement of the trained-SNHI centroid toward the NH group (Figure 7), which further could be attributed to the improved spatial performance in the trained-SNHI group, wherein spatial performance of a few individuals of this target group overlapped with the spatial skills of NH listeners leading to misjudgment of SNHI-trained as NH (which was not otherwise visible in pre-training evaluation phase).

## Conclusion

The findings from the current investigation highlighted the efficacy of VAST as an intervention tool for remediating spatial deficits in SNHI listeners. The success derived from VAST has promising implications for rehabilitative audiologists, as the tool has good clinical applicability. Although the VAST paradigm was done under laboratory conditions, it can also be adopted for spatial training at home as it requires only minimal equipment (laptop, paradigm player software, and headphones with good frequency response) at the user's end. The feasibility and applicability of the VAST paradigm with equipment already available at home make this protocol even more practical for implementation. The spatial training paradigm can also be extended to other clinical populations with spatial difficulties, such as individuals with central auditory processing disorder (CAPD), spatial processing disorders, and auditory neuropathy spectrum disorder (ANSD) after gathering research evidence. Future studies in this field should focus on the endurance of the learned capabilities over time, generalization limits, and the role of other cognitive factors in assessing the effects of VAST.

## Data availability statement

The data analyzed in this study will be made available by authors on request, for educational purposes. Requests should be directed to the corresponding author.

## Ethics statement

The studies involving human participants were reviewed and approved by AIISH Ethical Committee for biobehavioral research. The patients/participants provided their written informed consent to participate in this study.

## Author contributions

KN: conceptualization of the study, formulation of the methods, data collection, data pruning and statistical analysis, and the original draft preparation. AU: conceptualization of the study, formulation of the method, supervision, and the original draft preparation. RK: writing and visual depiction of data. All authors contributed to the article and approved the submitted version.
